# What is the functional reach of wastewater surveillance for respiratory viruses, pathogenic viruses of concern, and bacterial antibiotic resistance genes of interest?

**DOI:** 10.1186/s40246-023-00563-8

**Published:** 2023-12-18

**Authors:** Kevin J. Sokoloski, Rochelle H. Holm, Melissa Smith, Easton E. Ford, Eric C. Rouchka, Ted Smith

**Affiliations:** 1 Department of Microbiology and Immunology, School of Medicine, University of Louisville, 505 S. Hancock St., Louisville, KY 40202 USA; 2 Center for Predictive Medicine for Biodefense and Emerging Infectious Disease, University of Louisville, 505 S. Hancock St., Louisville, KY 40202 USA; 3 Christina Lee Brown Envirome Institute, School of Medicine, University of Louisville, 302 E. Muhammad Ali Blvd., Louisville, KY 40202 USA; 4 Department of Biochemistry and Molecular Genetics, School of Medicine, University of Louisville, 580 S. Preston St., Louisville, KY 40202 USA; 5 KY INBRE Bioinformatics Core, University of Louisville, 522 E. Gray St., Louisville, KY 40202 USA

**Keywords:** Genomic surveillance, Molecular surveillance, Outbreak response, Reporting, Stakeholders, Wastewater

## Abstract

**Background:**

Despite a clear appreciation of the impact of human pathogens on community health, efforts to understand pathogen dynamics within populations often follow a narrow-targeted approach and rely on the deployment of specific molecular probes for quantitative detection or rely on clinical detection and reporting.

**Main text:**

Genomic analysis of wastewater samples for the broad detection of viruses, bacteria, fungi, and antibiotic resistance genes of interest/concern is inherently difficult, and while deep sequencing of wastewater provides a wealth of information, a robust and cooperative foundation is needed to support healthier communities. In addition to furthering the capacity of high-throughput sequencing wastewater-based epidemiology to detect human pathogens in an unbiased and agnostic manner, it is critical that collaborative networks among public health agencies, researchers, and community stakeholders be fostered to prepare communities for future public health emergencies or for the next pandemic. A more inclusive public health infrastructure must be built for better data reporting where there is a global human health risk burden.

**Conclusions:**

As wastewater platforms continue to be developed and refined, high-throughput sequencing of human pathogens in wastewater samples will emerge as a gold standard for understanding community health.

**Supplementary Information:**

The online version contains supplementary material available at 10.1186/s40246-023-00563-8.

## Background

Human pathogens, despite being intimately intertwined with our communities in terms of human health, are often considered separately from the standpoint of human health risk [8, 11, 12, 15]. Indeed, commensal pathogens with dysbiotic potential, pathogens of opportunity, and true pathogens circulate within developed and developing communities alike, negatively impacting human health. Furthermore, the impact of pathogens on health continues to outpace human interventions, as antimicrobial resistance among bacteria, such as *Staphylococcus aureus*, *Escherichia coli*, *Streptococcus pneumoniae*, *Klebsiella pneumoniae*, *Pseudomonas aeruginosa*, and *Neisseria gonorrheae*, and fungal pathogens, such as *Candida auris*, has sharply increased over the last several decades, leading to the emergence of multidrug-resistant pathogens [3, 6, 15]. In addition to the rise of antimicrobial-resistant bacteria, human and zoonotic viruses remain a significant threat to human health and can be surveilled in wastewater from livestock farms, wet markets, and their surrounding areas [19].

There is growing interest in whole-genome sequencing and metagenomics for public health surveillance [1, 7, 9]. Despite a clear appreciation of the impact of human pathogens on community health, efforts to understand pathogen dynamics within populations have traditionally followed a narrow, targeted, approach and relied on the deployment of specific molecular probes for quantitative detection or on clinical detection and reporting. Nonetheless, as established by recent targeted wastewater approach efforts examining Polio [14] and further proven by analysis of Severe Acute Respiratory Syndrome Coronavirus 2 (SARS-CoV-2) [18], wastewater-based epidemiology (WBE), which leverages high-throughput sequencing, is a robust and highly adaptable platform where specific pathogens, antimicrobial resistance genes, and viral pathogens may be simultaneously monitored. A mounting body of evidence indicates that pathogen-associated nucleic acids, or whole pathogens themselves, are secreted at highly variable rates from the body in urine and/or feces, and these nucleic acids can be reliably qualitatively and quantitatively detected using molecular biology approaches [2, 16]. Therefore, as wastewater represents a cross-section of the community served, for pathogens where there is evidence of urine or fecal shedding of pathogen-derived nucleic acids, there is an opportunity to use wastewater as an anonymous pooled sample for genomic surveillance to provide a broad-scale overview of circulating respiratory viruses, pathogenic viruses of concern, and bacterial antibiotic resistance genes concurrently. Despite requiring specialized equipment and expertise, high-throughput sequencing of wastewater samples for the specific purpose of broadly detecting human pathogens and antimicrobial resistance genes is essential to a holistic understanding of pathogen dynamics within a community. Nevertheless, translating wastewater sequencing data and WBE findings into meaningful public health actions requires the establishment of cooperative relationships among scientists, public health stakeholders, and the community.

The primary purpose of this article is to provide a broad overview and summary of a series of ongoing next-generation sequencing wastewater pathogen surveillance efforts at the University of Louisville and to provide insights into the lessons learned regarding the laboratory operations, transdisciplinary partnerships, and economy of scale of next-generation sequencing wastewater surveillance.

## Main text

### Laboratory operations

While wastewater sampling and analyses have been of interest from an environmental health standpoint for decades [13], recent global health events have re-established interest in using wastewater to monitor human pathogens in developed and developing communities. Researchers have learned how to sample for SARS-CoV-2 infection (e.g., the etiologic agent of COVID-19 disease), and the approach is the same for panpathogen genomics: a 125 ml raw influent wastewater sample with no preservative collected by wastewater utility personnel from a piped sewer network at a treatment plant, intermediary pump station, or in-network neighborhood location. This is different from a U.S. Environmental Protection Agency [17] wastewater regulatory sample, but the COVID-19 pandemic facilitated the building of relationships that enable wastewater for public health sampling and existing infrastructure that now universally applies to this new phase of wastewater genomics.

During the COVID-19 pandemic, high-throughput sequencing of wastewater collected from minor catchments and aggregate wastewater treatment facilities was invaluable for understanding the genetic diversity of the pathogen in the community [10, 18]. Just as quantitative real-time polymerase chain reaction (qRT‒PCR)-mediated detection enabled a quantitative assessment of prevalence in the community, high-throughput sequencing revealed specific insights into the basal genetic diversity of the pathogen as it traversed the population, the timing and emergence of variants of interest/concern, and the reemergence of strains with consequences to public health. Importantly, the genomic surveillance of the SARS-CoV-2 pandemic led to a broad interest in understanding the temporal-spatial dynamics of pathogens in a community using high-throughput sequencing platforms, as the information obtained during the SARS-CoV-2-focused efforts proved vital to coordinating infection control responses of public health stakeholders.

As the intensity of the COVID-19 pandemic waned, researchers saw an interest arise in adapting the lessons learned from wastewater high-throughput sequencing efforts to better inform community health outside the pandemic context. In 2022, the University of Louisville developed a list of human pathogens of interest for a wastewater panel (Additional file 1: Supplemental Table S1). Pathogens included as part of the analysis were either part of a commercially available respiratory panel or a result of consultation with a number of stakeholders, including the University of Louisville Center for Predictive Medicine, the Louisville Metro Department of Public Health and Wellness, the University of Louisville Hospital, and the Kentucky Department of Public Health. Pragmatic considerations such as genome size and cross-reactivity with nonpathogenic species were also part of the design process, which typically resulted in short gene products for bacterial species, toxin and pathogenetic factors for bacterial plasmids, and whole genomes for viral pathogens. Due to the prior establishment of meaningful cooperative and collaborative agreements between academic, private, and city government stakeholders during the SARS-CoV-2 outbreak, the existing high-throughput sequencing efforts were readily able to qualitatively assess human pathogens using a tried-and-true wastewater analysis pipeline (*pun intended*).

The adaptation of high-throughput sequencing to qualitatively assess nucleic acid signatures of other human pathogens in wastewater samples has both solved and created new challenges. Foremost, sample concentration and enrichment through either PEG-assisted precipitation or aerogel capture is still necessary to assess anything other than the highly abundant wastewater-associated bacterial species. As high-throughput sequencing seeks to identify and compare nucleic acid sequences to whole reference genomes or cassettes of interest, the molecular target size for the detection of a given pathogen is much greater than that afforded by PCR-based detection modalities. In addition, as next-generation sequencing-based detection does not strictly rely on the use of target-specific primers and probes, the potential that a given pathogen remains undetected due to nucleic acid fragmentation or loss of integrity or the presence of sequence polymorphisms that interfere with primer or probe annealing is eliminated. Thus, high-throughput sequencing has a substantial advantage regarding the number of chances at detecting any given pathogen in a complex mixture. Nonetheless, challenges remain in the detection of a broad panel of pathogens in wastewater using a “one size fits all” or “one pot” approach that does not focus on specific pathogens of interest or consider the potential consequences of including (or rather failing to exclude) sequences known to be at excessive abundances.

During the establishment of the panpathogen wastewater surveillance early experiences at the University of Louisville, it became readily apparent that fecal shedding bacterial pathogens and associated antimicrobial resistance cassettes/genes were easier to detect than viral respiratory pathogens. A review of our collected wastewater data in conjunction with information provided by the local public health department revealed biases in detection arising from (i) the likelihood of incidence in the community and (ii) the fact that respiratory viruses are not shed in feces/urine at the same rates as gut bacteria carrying targeted toxins or antimicrobial content or other enteric viruses [2, 4]. For example, gut-associated bacteria such as *Pseudomonas*, *Enterococcus*, and *Acinetobacter* spp. were often among the highest detected sequences in terms of percent sequence coverage and depth of coverage. The overabundance of these sequences led us to classify these signals as “high-expected,” as they were highly represented in the dataset and reasonably anticipated, a priori, to be present in any given wastewater sample. A negative impact of including high-expected pathogens in wastewater surveillance efforts is that pathogens known to be in relatively high clinical prevalence in the community but not necessarily shed at high concentrations into community wastewater (such as respiratory syncytial virus, as per the available clinical data for the city of Louisville) were underrepresented in our sequencing efforts due to the overconsumption of available sequencing capacity by the high-expected pathogens. Pathogens such as these were classified as “low-expected.” As such, in future work, it may be advantageous to split the samples into groups of pathogens that are anticipated to have high- or low-expected signals (in terms of sequence coverage and depth) to enhance the limit of detection.

As reasonably anticipated from the fields’ experience with assessing SARS-CoV-2 prevalence using wastewater analysis, the depth of coverage and, in general, the sequence representation of a specific pathogen is likely to be dependent on the relative burden of that pathogen in the wastewater sample [10, 18]. For a given pathogen, the detection of contiguous sequences enables a degree of confidence in the detection of the pathogen, the overall coverage and depth of coverage correlate with prevalence in the community, allowing for semiquantitative inferences to be made on a week-to-week basis within an individual community sampled area without accompanying PCR or RT‒PCR quantification. With next-generation sequencing approaches, there is a concern regarding off-target hits arising from closely related pathogens or evolutionarily conserved sequences within pathogen families; however, a major strength of high-throughput sequencing-based approaches is the capacity to review the resulting genomic data to gain confidence in, or refute, the detection event by comparing the sequence information to databases of known sequence data, such as those curated by the National Library of Medicine National Center for Biotechnology Information (NCBI). However, the precise relationship between sequence coverage and incidence in the population remains to be fully understood and likely varies across individual pathogens based on relative presence/concentration, the molecular nature of the pathogen itself and how the rest of the targeted pathogens flux considering we have a total maximum sequencing depth (100 M reads) per run, so if one signal goes up, another signal must invariably go down. It is a balance between budgetary restrictions and the detection limit. This general concept of how many infected individuals is needed to derive a threshold for genuine detection, or more specifically how many individual excretion contributors are needed to impact the signal-to-noise ratio within wastewater genomics, remains at the forefront of many wastewater analysis efforts and is an area of ongoing interest for many similar efforts worldwide. It is likely that empirical observations for each pathogen will be needed to define specific limits of detection; however, as the rate and extent (e.g., number of particles/amounts of nucleic acid) of shedding undoubtedly differ from person to person and shedding rates vary with respect to time, as evidenced by SARS-CoV-2 [2], highly accurate estimates of incidence and prevalence are likely to be unattainable in the short term.

### Public health partnership and reporting

The benefit of the broad-spectrum perspective is to change from a narrow vision of notifiable diseases and instead to utilize wastewater as an early disease warning for a community. For deep sequencing of wastewater to be impactful data for real-time public health action, there is a need to prioritize a detailed understanding of the wastewater data to hospitalization burden in the local health care system; this may additionally vary based on regional environmental health factors as triggers. In the United States, the National Notifiable Disease Surveillance System tracks select infectious diseases using health professional reporting [5] but excludes many respiratory viruses, pathogenic viruses of concern, and bacterial antibiotic resistance genes of interest. For wastewater monitoring to support public health, ground truth clinical data will always be required for reference and calibration. A more inclusive public health infrastructure must be built for better data reporting across a global human health risk burden. Reporting methods for respiratory viruses, bacterial pathogens and bacterial antimicrobial resistance genes are also not well standardized, and whether the relative presence and absence of qualitative information is strong enough to elicit a public health response warrants further debate. Advocacy to standardize the reported quantification of antibiotic resistance genes in environmental samples has just recently started [20]. Furthermore, the Centers for Disease Control and Prevention National Wastewater Surveillance System (NWSS) plans to expand to other targets on the PCR platform, but national panpathogen reporting methods for wastewater should be discussed in parallel. To date, we have reported sequencing data on presence and absence to public health stakeholders in a short PowerPoint presentation weekly.

Another variable constraint is the frequency of sampling and the time-to-data required for meaningful impact on public health. While sampling frequency has been shown, for SARS-CoV-2 at least, to not significantly contribute to detection [18], other pathogens or emergence events may be impacted by sample timing. Second, data availability is a major factor influencing public health stakeholder decisions. The availability of wastewater sequencing data is dependent on the existence of the necessary instrumentation and personnel. With limitless resources, the minimal time from sample collection to data analysis and reporting could be within 3 days. Nonetheless, as the instrumentation required for wastewater surveillance via next-generation sequencing constitutes a considerable investment, it is often shared with other academic or research endeavors, resulting in longer timelines. With the resources currently available to our group, we assess and interpret wastewater data within 2 weeks. Our rate-limiting factors are molecular enrichment and sequencing run times. Regardless, whether an increased frequency of sample collection, preparation, and hastened analyses will lead to healthier communities has yet to be demonstrated, and building a comprehensive network of pathogen surveillance is more meaningful at present.

Throughout the COVID-19 pandemic, stakeholders have included health care providers, local health departments, and state and federal agencies, spanning disciplines from government to academia. Insights for public health action can be drawn from COVID-19 in that wastewater elicited a multidisciplinary team requiring partnership at a local level with a sewer utility provider, an analysis laboratory, and subject matter experts for data interpretation across these stakeholders. For other infectious diseases, this framework might need to be adapted; the opportunity for human and animal health experts remains [19]. Where there are multiple targets for wastewater screening focus, the process of defining stakeholders may be more complex than what has previously worked.

### Economics of scale

Individual testing provides individuals with information about their own health that cannot be achieved through WBE. Additionally, the public health surveillance system is often comprised of many pathogen-specific platforms that operate without interconnectivity; these have included testing drinking water, swimming pools, foods, sentinel animal species, and field-collected insects, alongside a robust clinical reporting channel such as the National Notifiable Disease Surveillance System [5]. Using the wastewater matrix as a platform to deploy broad screening tools such as genomic sequencing introduces significant economies of scale to the extent that a few microliters of nucleic acid from raw wastewater samples collected from a portion of the community can yield insights across many pathogen targets with geospatial and temporal context. We now have abundant evidence that a single wastewater sample can reflect a multitude of disease risks and passively capture many individuals’ health in the community with one sample. It is difficult to estimate the true economic value of this kind of broad-spectrum screening testing because it is unlikely that clinical testing of communities would ever be conducted at the scale witnessed for COVID-19 for each individual target—yet genomic analysis of wastewater samples can deliver this scale of testing at a marginal cost over the testing for a single pathogen. With the proper resources, including automation, a goal for the future of this field would be to have a cost-effective sequencing platform on a chip that could rapidly and robustly detect and characterize pathogens within 2–3 days, with easily customizable targets and standardized reporting. While this goal will take considerable effort and time to realize, the importance of wastewater pathogen surveillance to local and global public health demands that resources be allocated to meet this challenge.

During the COVID-19 pandemic, public health responses and scientific research received large investments from private and federal sources. As this robust support comes to an end, the Bipartisan Infrastructure Law (BIL), which includes $43.4 billion through the State Revolving Funds for the USA, may continue to help communities improve their water and wastewater infrastructure. However, how the resources afforded by the BIL apply to WBE is still undetermined. It may be beneficial to use the BIL to include planned infrastructure improvements to also allow for future public health monitoring, such as convenient system composite sampling access points, which might be done during construction. Largely, this funding is provided as grants or principal forgiveness loans to disadvantaged communities through Clean Water State Revolving Funds and Drinking Water State Revolving Funds. It would be advantageous to include WBE as a transdisciplinary component of the BIL to support the health of disadvantaged communities.

## Conclusion

Wastewater sequencing efforts continue to be a leading approach toward understanding pathogen incidence and diversity within a population. This strategy enables the qualitative and semiquantitative assessment of pathogens of interest across kingdoms. As development and refinement of the platform continues, high-throughput sequencing of human pathogens in wastewater samples will emerge as a gold-standard diagnostic of community health. Future directions include the need to overcome the challenges posed by diversity in microbial communities and how shifting of the panopoly of diverse organisms influences pathogen detection limits, defining transdisciplinary public health partnerships across multiple targets and working with public health and clinical partners to develop case studies of wastewater genomic sequencing leading to a public health outbreak response.

### Supplementary Information

**Additional file 1. Table S1: **List of sequences represented in the respiratory and panpathogen (N = 108) wastewater panels at the University of Louisville. Note that Klebsiella and Shewanella were removed due to their dominance in the overall reads.

## Data Availability

Data sharing is not applicable to this article as no datasets were generated or analyzed during the current study.

## Abbreviations

BIL: Bipartisan infrastructure law
COVID-19: Coronavirus disease 2019
qRT-PCR: Quantitative real-time polymerase chain reaction
SARS-CoV-2: Severe acute respiratory syndrome coronavirus 2
WBE: Wastewater-based epidemiology
