# Supplementary material for: What is the functional reach of wastewater surveillance for respiratory viruses, pathogenic viruses of concern, and bacterial antibiotic resistance genes of interest?
Source: Hum Genomics. 2023 Dec 18;17:114. doi: 10.1186/s40246-023-00563-8 (PMC10726489; doi:10.1186/s40246-023-00563-8)
Supplement: Supplementary file 1 — Additional file 1. Table S1: List of sequences represented in the respiratory and panpathogen (N = 108) wastewater panels at the University of Louisville. Note that Klebsiella and Shewanella were removed due to their dominance in the overall reads. [file 40246_2023_563_MOESM1_ESM.docx]

Supplemental Material

**What is the functional reach of wastewater surveillance for respiratory viruses, pathogenic viruses of concern, and bacterial antibiotic resistance genes of interest?**

Kevin J. Sokoloski^1,2†^, Rochelle H. Holm^3*†^, Melissa Smith^4^, Easton E. Ford^1^, Eric C. Rouchka^4,5^ and Ted Smith^3^

^1^Department of Microbiology and Immunology, School of Medicine, University of Louisville, 505 S. Hancock St., Louisville, KY 40202, United States

^2^Center for Predictive Medicine for Biodefense and Emerging Infectious Disease, University of Louisville, 505 S. Hancock St., Louisville, KY 40202, United States

^3^Christina Lee Brown Envirome Institute, School of Medicine, University of Louisville, 302 E. Muhammad Ali Blvd., Louisville, KY 40202, United States

^4^Department of Biochemistry and Molecular Genetics, School of Medicine, University of Louisville, 580 S. Preston St., Louisville KY 40202, United States

^5^KY INBRE Bioinformatics Core, University of Louisville, 522 E. Gray St., Louisville, KY 40202, United States

^*^Corresponding author: Rochelle H. Holm (rochelle.holm@lousiville.edu)

^†^These authors contributed equally and are considered co-first authors

**Table S1: List of sequences represented in the respiratory and panpathogen (N = 108) wastewater panels at the University of Louisville.** Note that Klebsiella and Shewanella were removed due to their dominance in the overall reads.

| **Accession** | **Description** |
| --- | --- |
| AC 000018.1 | Adenovirus 7 |
| AE001584.1 | Borrelia burgdorferi B31 plasmid lp56 |
| AF065404.1 | Bacillus anthracis strain Sterne plasmid pX01 |
| AF074611.1 | Yersinia pestis KIM10+ plasmid pMT-1 |
| AY524989.1 | Pseudomonas aeruginosa VIM-10 blaVIM-10 gene |
| JN032132.1 | Adenovirus 14 |
| JX869059.2 | Betacoronavirus 2c EMC-2012 |
| MG953830.1 | Bocavirus 1 |
| NC_000955.2 | Borreliella burgdorferi B31 plasmid lp21 |
| NC_001474.2 | Dengue virus 2 |
| NC_001489.1 | Hepatitis A |
| NC_001498.1 | Measles |
| NC_001542.1 | Rabies |
| NC_001545.2 | Rubella |
| NC_001560.1 | Vesicular stomatitis Indiana virus |
| NC_001563.2 | West Nile virus lineage 2 |
| NC_001617.1 | Rhinovirus A |
| NC_001672.1 | Tick-borne encephalitis virus |
| NC_001781.1 | Orthopneumovirus |
| NC_001796.2 | Respirovirus 3 |
| NC_001802.1 | Human immunodeficiency virus 1 |
| NC_001803.1 | Respiratory syncytial virus |
| NC_002058.3 | Poliovirus |
| NC_002200.1 | Mumps orthorubulavirus |
| NC_002204.1 | Influenza B |
| NC_002205.1 | Influenza B segment2 |
| NC_002206.1 | Influenza B segment3 |
| NC_002207.1 | Influenza B segment4 |
| NC_002208.1 | Influenza B segment5 |
| NC_002209.1 | Influenza B segment6 |
| NC_002210.1 | Influenza B segment7 |
| NC_002211.1 | Influenza B segment8 |
| NC_002645.1 | Coronavirus 229E |
| NC_003266.2 | Adenovirus E |
| NC_003443.1 | Rubulavirus 2 |
| NC_003461.1 | Respirovirus 1 |
| NC_003687.1 | Powassan virus |
| NC_003899.1 | Eastern equine encephalitis virus |
| NC_003977.2 | Hepatitis B virus strain ayw |
| NC_003985.1 | Porcine teschovirus 1 |
| NC_004108.1 | La Crosse virus segment L |
| NC_004109.1 | La Crosse virus segment M |
| NC_004110.1 | La Crosse virus segment S |
| NC_004162.2 | Chikungunya virus |
| NC_004718.3 | SARS coronavirus Tor2 |
| NC_005215.1 | Sin Nombre virus segment M |
| NC_005216.1 | Sin Nombre virus segment S |
| NC_005217.1 | Sin Nombre virus map viral genome L segment |
| NC_005236.1 | Seoul virus strain 80-39 segment S |
| NC_005237.1 | Seoul virus segment M |
| NC_005238.1 | Seoul virus strain Seoul 80-39 clone 1 |
| NC_005831.2 | Coronavirus NL63 |
| NC_006007.1 | Bluetongue virus segment 8 |
| NC_006008.2 | Bluetongue virus gene for VP6 protein |
| NC_006010.1 | Bluetongue virus segment 6 |
| NC_006013.2 | Bluetongue virus VP2 gene |
| NC_006014.1 | Bluetongue virus segment 3 |
| NC_006015.1 | Bluetongue virus segment 10 |
| NC_006022.1 | Bluetongue virus segment 7 |
| NC_006023.1 | Bluetongue virus segment 1 |
| NC_006024.2 | Bluetongue virus segment 4 |
| NC_006025.1 | Bluetongue virus segment 5 |
| NC_006213.1 | Coronavirus OC43 |
| NC_006577.2 | Coronavirus HKU1 |
| NC_007366.1 | H3N2 segment4 |
| NC_007367.1 | H3N2 segment7 |
| NC_007368.1 | H3N2 segment6 |
| NC_007369.1 | H3N2 segment5 |
| NC_007370.1 | H3N2 segment8 |
| NC_007371.1 | H3N2 segment3 |
| NC_007372.1 | H3N2 segment2 |
| NC_007373.1 | H3N2 segment1 |
| NC_007580.2 | Saint Louis encephalitis virus |
| NC_009495.1 | Clostridium botulinum A str ATCC 3502 GENE bont |
| NC_009996.1 | Rhinovirus C |
| NC_011203.1 | Adenovirus B1 |
| NC_012532.1 | Zika virus |
| NC_021928.1 | Parainfluenza virus 4a |
| NC_026431.1 | H1N1 segment7 |
| NC_026432.1 | H1N1 segment8 |
| NC_026433.1 | H1N1 segment4 |
| NC_026434.1 | H1N1 segment6 |
| NC_026435.1 | HIN1 segment2 |
| NC_026436.1 | H1N1 segment5 |
| NC_026437.1 | HIN1 segment3 |
| NC_026438.1 | H1N1 segment1 |
| NC_032093.1 | Candida albicans SC5314 GENE ERG11 |
| NC_032094.1 | Candida albicans SC5314 GENE MDR1 |
| NC_038308.1 | Enterovirus 68 |
| NC_038312.1 | Rhinovirus B3 |
| NC_038882.1 | Hepatitis C virus isolate H77 genotype 1 |
| NC_039199.1 | Metapneumovirus |
| NC_044946.1 | African swine fever virus strain Ken06 Bus |
| NC_045512.2 | SARS-CoV-2 |
| NC_063383.1 | Monkeypox |
| NG_047946.1 | Staphylococcus aureus MecA |
| NG_047955.1 | Staphylococcus aureus M10 0061 MecC |
| NG_048325.1 | Enterococcus faecium C864 vanA |
| NG_048341.1 | Enterococcus faecalis pMG2200 vanB |
| NW_021640162.1a | Candida auris strain B11221 GENE FKS1 |
| NW_021640162.1b | Candida auris strain B11221 GENE FKS1 |
| NZ_AP022255.1 | Aeromonas caviae strain WP8-S18-ESBL-04 plasmid pWP8-S18-ESBL-04 GENE H7R77 RS21020 |
| NZ_CP009716.1 | Corynebacterium ulcerans strain 05146 GENE tox |
| NZ_CP030031.1 | Acinetobacter radioresistens strain LH6 GENE DOM24 RS04840 |
| NZ_CP043954.1 | Acinetobacter baumannii strain K09-14 plasmid pK09-14 GENE F3P16 RS18800 |
| NZ_CP045197.1 | Acinetobacter indicus strain TQ23 plasmid p23TQ-NDM GENE GA693 RS00425 |
| NZ_CP071767.1 | Acinetobacter towneri strain GX3 plasmid pGX3-1 GENE J4G44 RS13030 |
| S71932.1 | Serratia marcescens imipenem resistant |
